# Severe symptomatic hypocalcemia with probable transient parathyroid dysfunction following radioiodine therapy for Graves’ disease: a case report

**DOI:** 10.1093/omcr/omag161

**Published:** 2026-09-02

**Authors:** Anamarija Jankulovska, Natasha Stojanoska, Tanja Makazlieva, Nevena Manevska, Sinisha Stojanoski

**Affiliations:** Institute of Pathophysiology and Nuclear Medicine "Acad Isac S. Tadzer", Faculty of Medicine, University of “Ss. Cyril and Methodius” in Skopje, Str. Mother Teresa 17, 1000 Skopje, Republic of North Macedonia; Institute of Histology and Embryology, Faculty of Medicine, University of “Ss. Cyril and Methodius” in Skopje, Str. Mother Teresa 17, 1000 Skopje, Republic of North Macedonia; Institute of Pathophysiology and Nuclear Medicine "Acad Isac S. Tadzer", Faculty of Medicine, University of “Ss. Cyril and Methodius” in Skopje, Str. Mother Teresa 17, 1000 Skopje, Republic of North Macedonia; Institute of Pathophysiology and Nuclear Medicine "Acad Isac S. Tadzer", Faculty of Medicine, University of “Ss. Cyril and Methodius” in Skopje, Str. Mother Teresa 17, 1000 Skopje, Republic of North Macedonia; University Institute of Positron Emission Tomography, Bledski Dogovor 10, 1000 Skopje, Republic of North Macedonia; Institute of Pathophysiology and Nuclear Medicine "Acad Isac S. Tadzer", Faculty of Medicine, University of “Ss. Cyril and Methodius” in Skopje, Str. Mother Teresa 17, 1000 Skopje, Republic of North Macedonia

**Keywords:** radioiodine therapy, hypocalcemia, vitamin D deficiency, parathyroid dysfunction

## Abstract

**Background:**

Radioactive iodine therapy (RAIT) is a definitive treatment for hyperthyroidism, but parathyroid effects are often underrecognized.

**Case Presentation:**

A 66-year-old woman with a 30-year history of Graves’ disease underwent RAIT (555 MBq ^131^I) after long-term treatment with thiamazole and briefly with propylthiouracil without achieving remission. Two weeks after RAIT, she developed paresthesia and muscle cramps, progressing to hospitalization one week later with positive Chvostek’s and Trousseau’s signs. Laboratory tests showed severe hypocalcemia (ionized calcium 0.70 mmol/L), vitamin D deficiency, and low parathyroid hormone levels, consistent with parathyroid dysfunction (CTCAE Grade 3 hypocalcemia). She required intravenous and oral calcium plus vitamin D, with gradual improvement. At 9-month follow-up, calcium levels remained stable on supplementation, suggesting prolonged recovery of parathyroid function. She later developed post-RAIT hypothyroidism.

**Conclusion:**

This case highlights the importance of considering transient parathyroid dysfunction in patients with symptomatic hypocalcemia after RAIT and supports long-term biochemical monitoring and supplementation.

## Introduction

Graves’ disease (GD) is the most common cause of hyperthyroidism, affecting approximately 3% of women and 0.5% of men during their lifetime [1]. Radioactive iodine therapy (RAIT) is a well-established and highly effective definitive treatment for GD, achieving durable remission in most patients with thyrotoxicosis and demonstrating an excellent safety profile in both benign and malignant thyroid disease [2]. Although the thyroid is the primary target, the maximum beta-particle range of 2.4 mm, combined with the close anatomical proximity of the parathyroid glands, may result in incidental radiation exposure to these glands [3].

Common adverse effects of RAIT are well recognized and include radiation thyroiditis, transient worsening of hyperthyroidism, salivary gland dysfunction, xerostomia, and worsening of thyroid eye disease. However, parathyroid dysfunction is not addressed in current guidelines and remains insufficiently investigated [2, 4]. This may reflect its relative rarity, often transient nature, and the lack of systematic monitoring in routine clinical practice. The absence of routine calcium and parathyroid hormone (PTH) assessment after RAIT may therefore delay identification of affected patients, contributing to underdiagnosis and potential underestimation of its true incidence and clinical significance [5].

This case describes severe post-radioiodine hypocalcemia associated with vitamin D deficiency and corticosteroid therapy, underscoring transient parathyroid dysfunction as a potential adverse effect of radioiodine treatment.

## Case presentation

A 66-year-old woman with a 30-year history of GD, treated long-term with high-dose thiamazole and briefly with propylthiouracil without achieving remission, presented for definitive therapy. She had persistently suppressed TSH and elevated TRAb levels (up to 30 IU/L). Over the past six years, she developed progressive thyroid-associated ophthalmopathy, with mild-to-moderate right-sided exophthalmos. Her comorbidities included diabetes mellitus, chronic obstructive pulmonary disease, and hypertension.

Thyroid ultrasonography demonstrated hypoechoic, heterogeneous, hypervascular thyroid tissue and a 14 mm right-lobe nodule with benign cytology (Bethesda II on repeated fine-needle aspiration). Due to persistent hyperthyroidism, clinical deterioration, and refusal of surgery, the patient underwent empirically selected radioiodine therapy (^131^I, 15 mCi), followed by thiamazole 20 mg daily, pantoprazole 40 mg daily, and oral prednisone (40 mg daily, tapered over 30 days). No acute adverse effects were observed during the first few days after RAIT.

**Figure 1:**
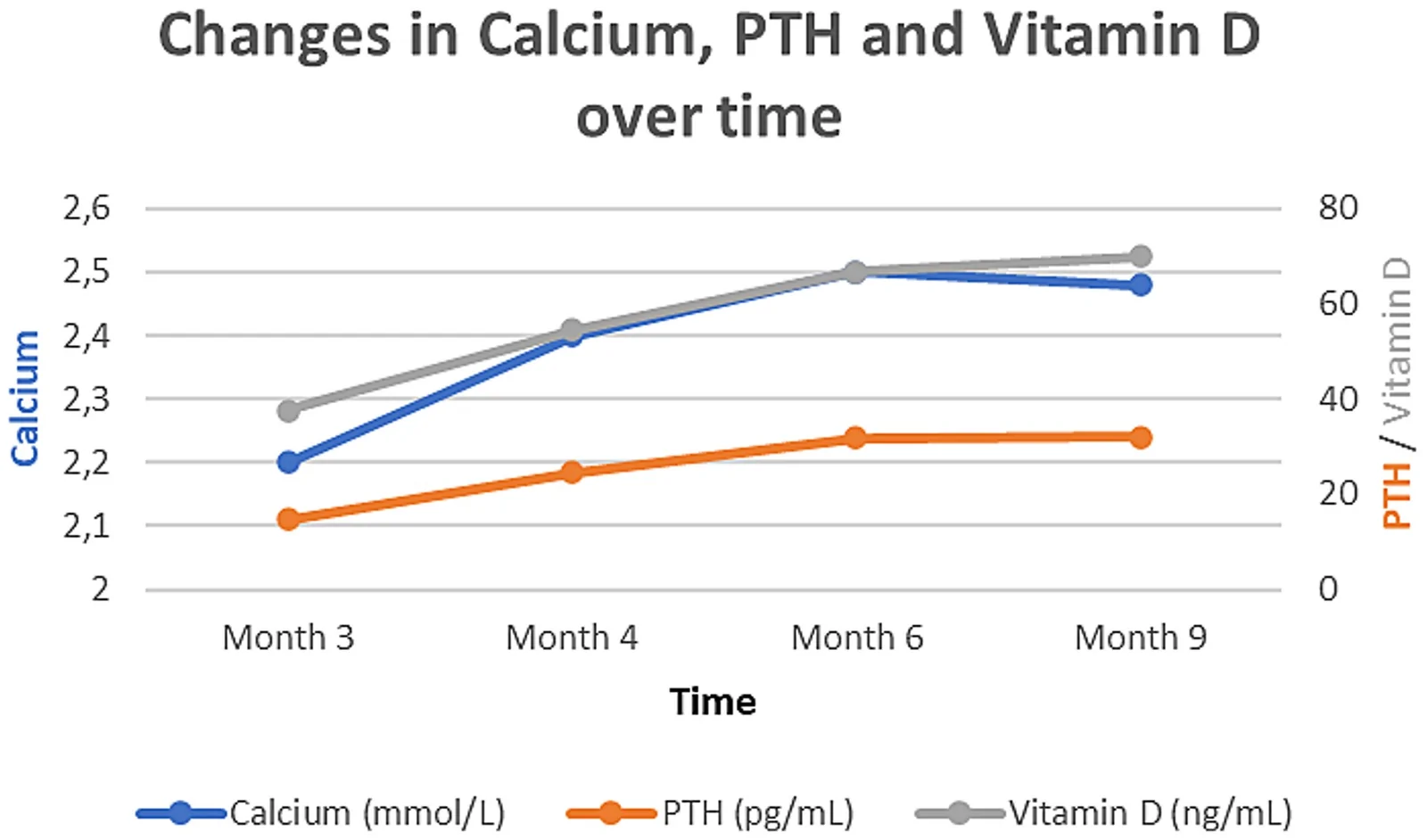
Serial biochemical changes following radioiodine therapy demonstrating gradual recovery of calcium, vitamin D, and parathyroid hormone (PTH) levels under continued calcium and vitamin D supplementation.

Two weeks after therapy, the patient developed paresthesia of the hands, jaw, and lower extremities, progressing to muscle cramps. Total serum calcium was 1.4 mmol/L, and she was treated for one week in a local general hospital with intravenous calcium gluconate (2 g daily), oral calcium citrate (1000 mg daily), and vitamin D (4000 IU daily). Three weeks after RAIT, she was hospitalized at the endocrinology clinic due to persistent symptoms. Physical examination revealed positive Chvostek’s and Trousseau’s signs. Laboratory evaluation confirmed CTCAE Grade 3 hypocalcemia, with ionized calcium of 0.70 mmol/L, PTH 10.8 pg/mL, vitamin D deficiency (13.83 ng/mL), and phosphate of 1.1 mmol/L. During the one-week hospitalization, serial ionized calcium measurements demonstrated gradual normalization (0.70, 0.83, 0.86, 1.14, and 1.18 mmol/L) following intravenous and oral calcium supplementation combined with vitamin D therapy. At discharge, calcium and vitamin D supplementation were continued. Thiamazole was gradually tapered and discontinued after 3 months. Levothyroxine replacement was initiated at 4 months and progressively increased to 100 μg daily by month 9. During follow-up, biochemical monitoring demonstrated gradual normalization of calcium, vitamin D, and PTH levels, suggesting recovery of parathyroid function (Fig. 1). Clinical and biochemical follow-up are summarized in Table 1.

**Table 1:** Clinical course, biochemical findings, and treatment timeline.

Time point	Clinical event	Laboratory findings	Treatment/intervention
Baseline (pre-RAIT)	Long-standing Graves’ disease, mild-to-moderate right-sided exophthalmos; refusal of surgery	Persistently suppressed TSH; elevated TRAb (up to 30 IU/L)	Long-term high-dose thiamazole; brief propylthiouracil therapy
Pre-RAIT evaluation	Thyroid ultrasound showed a mildly enlarged right lobe and a hypoechoic, heterogeneous gland with increased vascularity and a 14 mm hyperechoic nodule.	Bethesda category II cytology	Decision to proceed with definitive RAIT
Day 0	RAIT administered	—	^131^I therapy, 555 MBq (15 mCi); post-therapy treatment with thiamazole 20 mg daily, pantoprazole 40 mg daily, and oral prednisone (40 mg daily, tapered over 30 days)
Week 2 after RAIT	Paresthesia of the hands, jaw, and lower extremities, progressing to muscle cramps	Total calcium 1.4 mmol/L (reference range, 2.1–2.6)	Treated at a local general hospital with calcium gluconate 2 g i.v. daily, calcium citrate 1000 mg daily, and vitamin D 4000 IU daily for one week
Week 3 after RAIT	Hospitalized at the endocrinology clinic; positive Chvostek’s and Trousseau’s signs; symptomatic hypocalcemia	Ionized calcium during hospitalization: 0.70 → 0.83 → 0.86 → 1.14 → 1.18 mmol/L; PTH 10.8 pg/mL (reference range, 12–88); vitamin D 13.83 ng/mL (reference range, 30–100); phosphate 1.1 mmol/L (reference range, 0.80–1.50)	Intravenous and oral calcium supplementation plus oral vitamin D during one-week hospitalization
At discharge	Clinical improvement with resolution of paresthesia and disappearance of signs of hypocalcemia	Near-normal calcium levels at discharge	Calcium carbonate 1000 mg twice daily, calcium citrate 1000 mg twice daily, vitamin D 4000 IU daily; thiamazole dose reduced to 10 mg daily due to FT4 decline
Month 2 after RAIT	Clinical stabilization; reduction in thyroid and nodule volume on ultrasound	—	Thiamazole reduced to 5 mg daily; continued calcium carbonate, calcium citrate, and vitamin D supplementation
Month 3 after RAIT	Clinically improved, on continued supplementation	Total calcium 2.2 mmol/L; vitamin D 23.0 ng/mL; PTH 14.7 pg/mL	Continued calcium carbonate 1000 mg twice daily, calcium citrate 1000 mg twice daily, vitamin D 4000 IU daily; thiamazole discontinued
Month 4 after RAIT	Stable under supplementation	Total calcium 2.4 mmol/L; vitamin D 30 ng/mL; PTH 24.5 pg/mL	Same supplementation continued; levothyroxine 50 μg daily initiated
Month 6 after RAIT	Clinically stable under ongoing supplementation	Total calcium 2.5 mmol/L; vitamin D 34 ng/mL; PTH 31.8 pg/mL	Continued calcium carbonate 1000 mg twice daily, calcium citrate 600 mg twice daily, vitamin D 4000 IU daily; levothyroxine increased to 75 μg daily
Month 9 after RAIT	Clinically stable	Mild TSH elevation suggestive of evolving post-radioiodine hypothyroidism	Levothyroxine increased to 100 μg daily; supplementation continued

## Discussion

Parathyroid dysfunction is an uncommon but clinically relevant complication of RAIT. Proposed mechanisms include direct β-radiation due to anatomical proximity, paracrine ‘bystander’ effects mediated by oxidative stress and cytokines, and transient post-radiation inflammation [3, 6, 7]. Additional factors, such as concomitant glucocorticoid therapy, may further increase hypocalcemia risk by impairing intestinal calcium absorption, enhancing vitamin D catabolism, and promoting urinary calcium loss. Furthermore, pre-existing vitamin D deficiency may predispose patients to clinically significant hypocalcemia following RAIT [8].

Clinically, parathyroid dysfunction after RAIT is usually transient, with symptoms appearing within 1 week to 3 months. Serum PTH levels may range from normal to inappropriately low, and hypocalcemia occurs in approximately 20% of patients. Spontaneous recovery or improvement with calcium and vitamin D supplementation is common, while persistent dysfunction is rare. Only 12 cases have been reported, including two pediatric patients; carpopedal spasms and paresthesia were the most frequent symptoms [8, 9]. One reported case involved a 12-year-old girl with GD, vitamin D deficiency, and asthma, who developed symptomatic hypocalcemia after RAIT (11.1 mCi). Laboratory evaluation revealed hypocalcemia, hyperphosphatemia, and inappropriately low-normal PTH, highlighting the importance of assessing and correcting vitamin D status prior to therapy [8].

Recent studies in DTC patients indicate that RAIT generally does not significantly alter calcium or PTH levels in individuals with normal baseline parathyroid function. Lower baseline calcium and PTH, as well as larger residual thyroid tissue, were identified as risk factors for transient hypocalcemia, typically resolving within six months [5, 6]. A recent study showed that higher RAI activities were associated with lower calcium and PTH at one year, though long-term differences were negligible, except in a single patient who developed normocalcemic hyperparathyroidism [10].

The development of probable transient parathyroid dysfunction in our patient was likely multifactorial, involving vitamin D deficiency, corticosteroid therapy, high local ^131^I uptake in hyperfunctioning thyroid tissue, and possibly increased bone turnover associated with GD. Although baseline vitamin D levels were unavailable, the inappropriately low PTH level despite severe hypocalcemia suggests that vitamin D deficiency alone is unlikely to explain the clinical presentation. The gradual recovery of PTH levels over nine months further supports reversible parathyroid dysfunction rather than permanent gland failure.

Although RAI-associated parathyroid dysfunction is biologically plausible, causality cannot be established with certainty in an individual patient. Based on this case and available literature, we suggest pre-RAI assessment and optimization of calcium and vitamin D status, with early post-therapy monitoring of calcium and PTH in high-risk patients.
